# Supplementary material for: Identification of photoperiod-induced specific miRNAs in the adrenal glands of Sunite sheep (Ovis aries)
Source: Front Vet Sci. 2022 Jul 22;9:888207. doi: 10.3389/fvets.2022.888207 (PMC9354845; doi:10.3389/fvets.2022.888207)
Supplement: Supplementary file 1 [file Data_Sheet_1.ZIP › Supplementary file/miRDeep2 file.zip!/miRDeep2 file/Know_Structure/SP42A1/oar-mir-148a.pdf]

miRBase precursor	: oar-mir-148a
Total read count	: 1858705
oar-miR-148a read count	: 1855324
remaining reads	: 3381

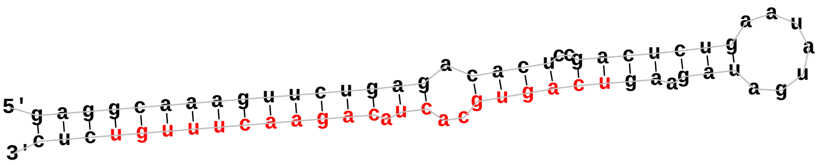

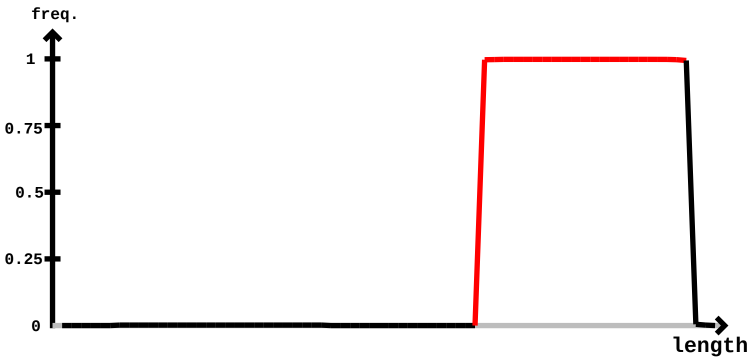

oar-miR-148a

5'- gaggcaaaguucugagacacuccgacucugaauaugauagaagucagugcacuacagaacuuugucuc -3' exp

((((((((((((((((.((((..(((((((.......))).))))))))..)).))))))))))))))	reads	mm	sample
...gcaaaguucugagacacuccgacu...	1	0	seq
....caaaguucugagacacuccga...	1	0	seq
....caaaguucugagacacuccgac..	1	0	seq
....caaaguucugagacacuccgacu...	69	0	seq
.....aaaguucugagacacuccg..	4	0	seq
.....aaaguucugagacacuccga...	79	0	seq
.....aaaguucugagacacuccgac..	35	0	seq
.....aaaguucugagacacuccgacu...	3177	0	seq
.....aaaguucugagacacuccgacuc..	3	0	seq
.....aaaguucugagacacuccgacucu.......................................	3	0	seq
......aaguucugagacacuccgacu...	8	0	seq
....gucagugcacuacagaacuu......	1	0	seq
....gucagugcacuacagaacuuug....	1	0	seq
....gucagugcacuacagaacuuugu...	63	0	seq
....guUagugcacuacagaacuuugu...	1	1	seq
....gucagugcacuacagaacuuuguc..	1	0	seq
....ucagugcacuacagaac........	5	0	seq
....ucagugAacuacagaacu.......	1	1	seq
....uUagugcacuacagaacu.......	1	1	seq
....ucagugcacuacagaacu.......	106	0	seq
....ucaCugcacuacagaacu.......	1	1	seq
....ucagugcacuacagaacuu......	251	0	seq
....ucagugcacuacagaacuuu.....	1809	0	seq
....uUagugcacuacagaacuuu.....	6	1	seq
....ucagugcacuacagaacuuug....	5598	0	seq
....uUagugcacuacagaacuuug....	13	1	seq
....uUagugcacuacagaacuuugu...	5051	1	seq
....ucagugcacuacagaacuuugu...	1831084	0	seq
....uUagugcacuacagaacuuuguc..	16	1	seq
....ucagugcacuacagaacuuuguc..	5521	0	seq
....ucagugcacuacagaacuuugucu.	3262	0	seq
....ucagugcacuacagaacuuugucuc	14	0	seq
....cagugcacuacagaacuuug....	2	0	seq
....Uagugcacuacagaacuuugu...	42	1	seq
....cagugcacuacagaacuuugu...	1005	0	seq

oar-miR-148a

```
gaggcaaaguucugagacacuccgacucugaauaugauagaagucagugcacuacagaacuuugucuc
............................................cagugcacuacagaacuuuguc..    5       0       seq
............................................cagugcacuacagaacuuugucu.    5       0       seq
.............................................agugcacuacagaacuuu.....    3       0       seq
.............................................agugcacuacagaacuuug....    5       0       seq
.............................................agugcacuacagaacuuugu...    1428    0       seq
.............................................agugcacuacagaacuuuguc..    4       0       seq
.............................................agugcacuacagaacuuugucu.    2       0       seq
..............................................gugcacuacagaacuuugu...    2       0       seq
...............................................ugcacuacagaacuuugu...    2       0       seq
...............................................ugcacuacagaacuuugucu.    1       0       seq
................................................gcacuacagaacuuugu...    12      0       seq
```
